# Screening for Peripheral Neuropathy and Peripheral Vascular Disease in Patients With Type 2 Diabetes Mellitus: A Community-Based Cross-Sectional Study From Rural Andhra Pradesh, India

**DOI:** 10.7759/cureus.93243

**Published:** 2025-09-25

**Authors:** Guna Sai Vallapuri, Sneha Udamala, Anil R, Siddharth Vallapuri, Amreen Vajrala

**Affiliations:** 1 Cardiology, Yashoda Super Speciality Hospitals, Hyderabad, IND; 2 Medical Education, PES Institute of Medical Sciences and Research, Kuppam, Kuppam, IND; 3 Preventive Medicine, PES Institute of Medical Sciences and Research, Kuppam, Kuppam, IND; 4 Medical Education, Gandhi Medical College, Hyderabad, IND

**Keywords:** community-based study, diabetic foot care, diabetic foot infection, diabetic peripheral neuropathy (dpn), peripheral motor neuropathy, peripheral sensory neuropathy, peripheral vascular disease (pvd), preventive health screening, rural health services, type 2 diabetes mellitus

## Abstract

Background

Diabetes mellitus continues to rise globally, with complications such as peripheral neuropathy and peripheral vascular disease (PVD) contributing substantially to morbidity, particularly in rural communities where awareness is low and care is inadequate.

Objective

To assess the burden of neuropathic symptoms and evaluate foot-care practices among adults with type 2 diabetes mellitus in rural Andhra Pradesh, India, with peripheral vascular status recorded qualitatively through pulse screening.

Methods

A cross-sectional community-based study was conducted across five villages. A total of 173 known type 2 diabetic patients aged ≥40 years were selected using random sampling. Data collection included interviews and clinical assessments for neuropathy and PVD.

Results

Peripheral neuropathic symptoms were highly prevalent, with 153 patients (88.44%) experiencing loss of hot/cold discrimination. A total of 130 patients (75.1%) had one or more comorbidities. While 164 patients (94.79%) were on treatment for diabetes, only 29 patients (16.8%) had undergone HbA1c testing in the last year. Foot-care practices were suboptimal - only six patients (3.5%) reported applying moisturiser between the toes, and just three patients (1.7%) used a mirror to inspect their feet regularly.

Conclusion

Peripheral complications and poor foot-care practices remain widespread. Strengthening education and early screening in rural settings is essential to curb diabetic foot morbidity.

## Introduction

Diabetes is a chronic metabolic disease characterised by persistent hyperglycaemia that leads to micro- and macro-vascular complications, notably neuropathy and peripheral arterial disease, which together drive diabetic foot risk. India has one of the highest global burdens of type 2 diabetes mellitus (T2DM). The Indian Council of Medical Research-India Diabetes (ICMR-INDIAB) study in 2011 reported over 62 million T2DM cases, projected to rise to 101 million cases by 2030 [1]. Despite its rising prevalence, diabetes is often under-reported as a cause of death [2]. One of the most disabling complications is diabetic foot disease, resulting from peripheral neuropathy and peripheral vascular disease (PVD), often leading to ulceration, infection, gangrene, and ultimately amputation [3]. Amputations secondary to diabetes occur somewhere in the world every 30 seconds due to diabetic foot complications [4].

India bears one of the world’s largest T2DM burdens, with rapid growth in rural districts where access to screening and education is limited; this context underscores the need for targeted diabetic foot prevention in underserved communities.

In India, data on foot complications among diabetics at the community level are scarce, particularly concerning neuropathy, vascular symptoms, and foot-care practices. In South India, the confluence of peripheral neuropathy and PVD substantially elevates the risk of ulceration and amputation, reinforcing the value of early foot-care education and screening. This study was therefore conducted to assess neuropathy, PVD, and foot-care awareness among diabetic patients in the field practice area of PES Institute of Medical Sciences and Research (PESIMSR), Kuppam, Andhra Pradesh, India.

## Materials and methods

This community-based cross-sectional study was conducted in five rural villages near PESIMSR, Kuppam, Andhra Pradesh. The study period was from December 2022 to January 2023. A total of 173 known T2DM patients aged ≥40 years were enrolled. From village line-lists of known T2DM patients, we used a computer-generated random number sequence to select approximately 34 participants per village. Inclusion criteria were adults aged ≥40 years with a known diagnosis of T2DM who were permanent residents of the study villages and who consented to participate. Exclusion criteria included unwillingness to provide consent, presence of an acute foot ulcer/infection at the time of survey, or severe illness precluding examination. Smoking history and prior exposure to foot-care education were recorded for analysis, while those aged <40 were excluded due to the lower prevalence of neuropathy and vascular disease in this age group. Ethical clearance was obtained from the Institutional Human Ethics Committee (PESIMSR/IHEC/158-22), and written informed consent was obtained from all participants.

**Figure 1:**
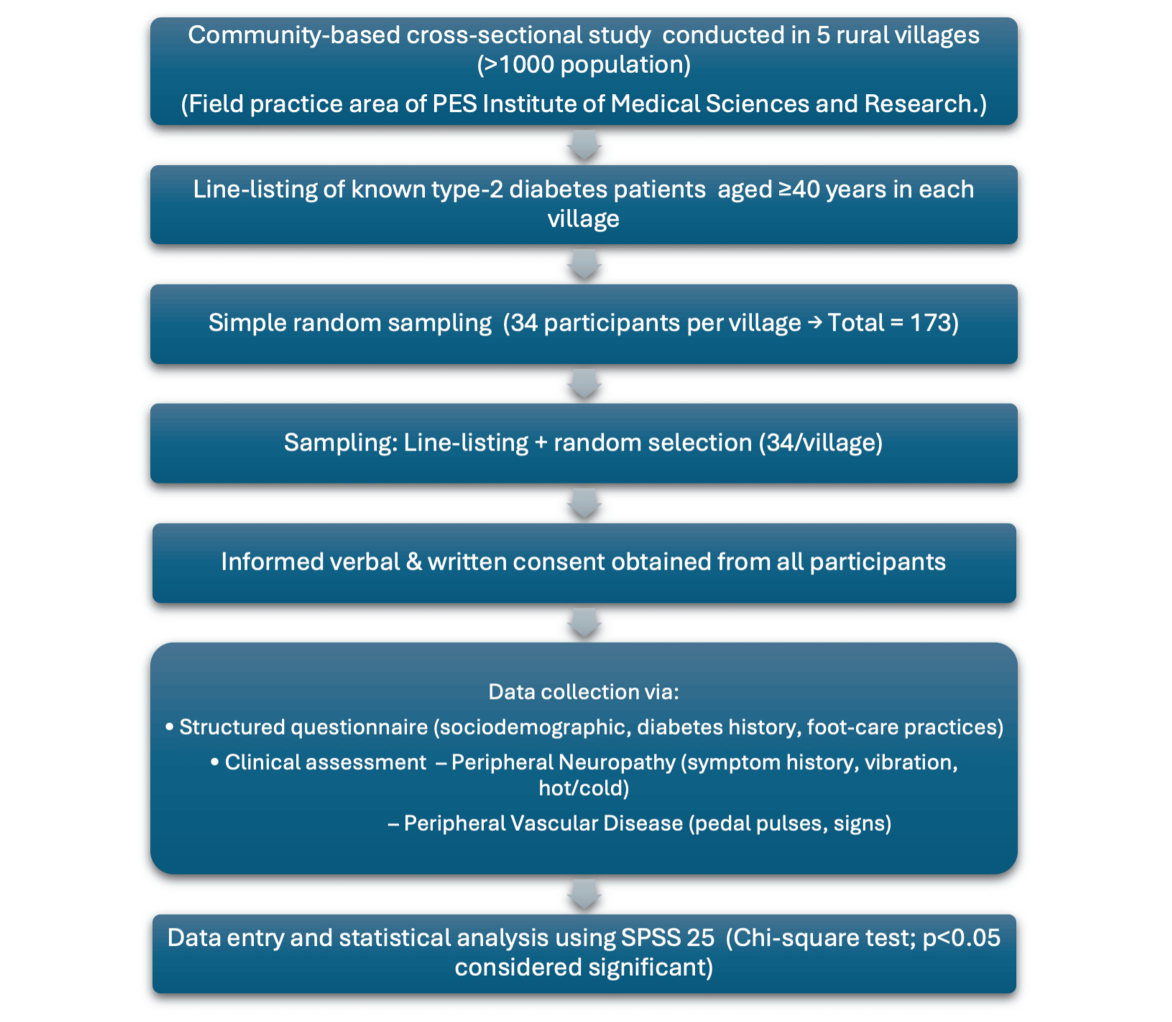
Flowchart Depicting the Methodology of the Study

The sample size (n=168; rounded to 173) was calculated based on previous prevalence estimates from South India [5]. We restricted enrolment to age ≥40 years because neuropathy risks and cumulative glycaemic exposure rise meaningfully after the fourth decade, aligning with our community screening focus. Data were collected by three trained investigators using a structured questionnaire covering demographics, medical history, neuropathic symptoms, and foot-care practices, followed by clinical examination (see Appendix A). Peripheral neuropathy was assessed using hot/cold discrimination, monofilament, and tuning fork tests, while PVD was assessed via palpation of dorsalis pedis (DP) and posterior tibial (PT) pulses. Anthropometric measurements and HbA1c levels were recorded. Data were entered in Microsoft Excel (Microsoft® Corp., Redmond, WA, USA) and analysed using IBM SPSS Statistics for Windows, Version 25 (Released 2017; IBM Corp., Armonk, New York, United States). Chi-square tests were used to assess associations between categorical variables, with p<0.05 considered statistically significant. The study methodology is summarised in Figure 1.

## Results

The socio‑demographic profile of the study population is shown in Table 1. As shown in Table 1, most participants were male (108, 62.42%), Hindu (143, 82.65%), and engaged in agriculture (66, 38.15%). Nearly half (82, 47.39%) belonged to class 4 socioeconomic status, and primary education (74, 42.77%) was the most common level attained. The high proportion of agricultural workers and the low literacy rate reflect the rural setting and suggest the need for accessible, tailored health education strategies.

**Table 1:** Socio-Demographic Profile of the Study Population

		Number (n)	Percentage (%)
Gender	Male	108	62.42
	Female	65	37.57
Religion	Hindu	143	82.65
	Christian	16	9.24
	Muslim	14	8.09
Occupation	Agriculture	66	38.15
	Homemaker	26	15.02
	Clerical/shop owner	20	11.56
	Professional	4	2.31
	Semi-professional	2	1.15
	Skilled	6	3.46
	Semi-skilled	9	5.20
	Unskilled	8	4.62
	Student	3	1.73
	Retired	9	5.20
	Unemployed	20	11.56
Education	Graduate	11	6.35
	Intermediate	10	5.78
	High school	15	8.67
	Middle school	33	19.07
	Primary school	74	42.77
	Illiterate	30	17.34
Marital status	Married	160	92.48
	Unmarried	10	5.78
	Divorced	2	1.15
	Widow/widower	1	0.57
Socio-economic status	Class 1	1	0.57
	Class 2	7	4.04
	Class 3	53	30.63
	Class 4	82	47.39
	Class 5	30	17.34

The clinical and investigation characteristics of the study population are presented in Table 2. Over half of participants (101, 58.4%) reported a family history of diabetes, and 98 (56.64%) had been diagnosed for one to five years. The majority were on treatment, 164 (94.79%), with 94 (54.3%) on oral hypoglycaemic drugs and 67 (38.72%) on a combination of oral agents and insulin. Only 29 (16.76%) had undergone HbA1c testing in the past year, as shown in Table 2.

**Table 2:** Clinical and Investigation Characteristics of the Participants HbA1c: glycated haemoglobin; FBS: fasting blood sugar; GRBS: general random blood sugar; PPBS: postprandial blood sugar

		Number (n)	Percentage (%)
Family history	Absent	72	41.61
	Maternal	63	36.41
	Paternal	25	14.45
	Siblings	13	7.51
Number of years of diabetes	<1 year	32	18.49
	1-5 years	98	56.64
	5-10 years	25	14.45
	>10 years	18	10.40
Treatment modality	Oral hypoglycaemic	94	54.33
	Insulin	3	1.73
	Both	67	38.72
	Dietary management	4	2.31
	None	5	2.89
Co-morbidities	Present	130	75.14
	Absent	43	24.85
HbA1c done in the past year	Yes	29	16.76
	No	144	83.23
FBS done in the past year	Yes	93	53.75
	No	80	46.24
GRBs done in the past year	Yes	136	78.61
	No	37	21.38
PPBS done in the past year	Yes	45	26.01
	No	128	73.98

**Figure 2:**
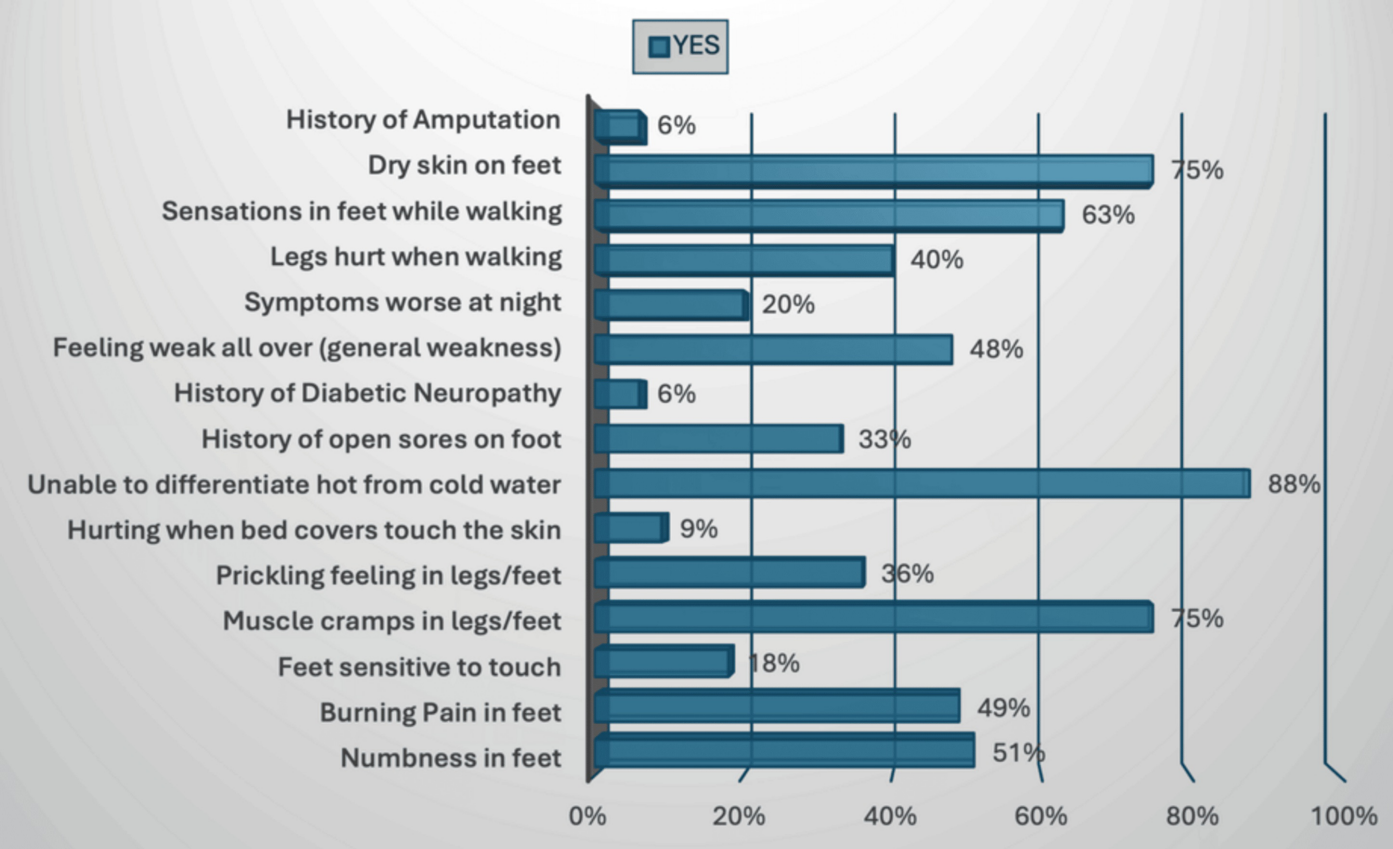
Distribution of Neuropathic Symptoms Among Study Participants Bars represent the proportion reporting each symptom (n = 173).

Figure 2 shows the distribution of neuropathic symptoms reported by participants. Impaired hot/cold sensation was the most common (153, 88.44%), followed by muscle cramps (130, 75.14%), dry skin (130, 75.14%), numbness (88, 50.87%), burning pain (85, 49.13%), and general weakness (83, 48.0%).

Peripheral vascular assessment (physical exam)

DP and PT pulses were examined in all participants as part of the study protocol. While the presence or absence of pulses was clinically assessed, the exact frequency of diminished or absent pulses was not systematically recorded. Therefore, a precise prevalence of PVD could not be calculated. This represents a limitation of the study and is acknowledged accordingly.

The foot‑care practices reported by participants are summarised in Table 3. Table 3 summarises foot-care practices, revealing that more than half (91, 52.60%) did not use moisturiser; 28 (19.04%) did not wipe their feet after washing; and only three (1.73%) used a mirror for self-inspection. Just 38 (21.96%) chose footwear suitable for their diabetic status. These findings point to inadequate awareness and practice of preventive foot care, despite the high risk of complications, highlighting a critical area for community-based education and intervention.

**Table 3:** Foot-Care Practices Reported by Participants

		Number (n)	Percentage (%)
Washing of legs and feet	Does not wash at all	26	15.02
	Washes with lukewarm water	135	78.03
	Washes with hot water	12	6.93
Wiping of legs and feet after washing	Doesn’t wipe at all	28	19.04
	Doesn’t wipe the web spaces	100	68.02
	Wipes the web spaces	19	12.92
Application of moisturiser	Applies in the web spaces	6	3.46
	Doesn’t apply at all	91	52.60
	Leaves out the web spaces	76	43.93
Use of mirror for foot examination	Yes	3	1.73
	No	170	98.26
Nail cutting	Cuts nails straight carefully	84	48.55
	Not careful while cutting nails	25	14.45
	Doesn’t use nail cutter at all	64	36.99
Footwear practice	Chooses the correct footwear	38	21.96
	Doesn’t choose the correct footwear	64	36.99
	No idea that the footwear is important	71	41.04

The association between participant characteristics and foot-care practices is detailed in Table 4. Foot-care practices were categorised into two groups based on overall behaviour. A subtle trend was noted with age, with foot-care habits declining as participants grew older (X^2^ = 7.75; p = 0.051). Males (65, 60.19%) were more likely to have poor practices than females (31, 47.69%), and the differences by religious affiliation likely reflect the area’s demographic composition and unmeasured social factors; we therefore refrain from inferring causality. None of these associations reached statistical significance.

**Table 4:** Association Between Participant Characteristics and Foot-Care Practices

Risk factor		<5 (Poor Foot-Care Practices)	>5 (Good Foot-Care Practices)	Total	Chi-Square (X^2^)	P-value
Age (years)	<40 years	7 (41.18%)	10 (58.82%)	17 (100%)	7.7540	0.051
	41-50 years	24 (43.64%)	31 (56.36%)	55 (100%)		
	51-60 years	38 (64.41%)	21 (35.59%)	50 (100%)		
	>60 years	27 (64.29%)	15 (35.71%)	42 (100%)		
	Total	96 (55.49%)	77 (44.51%)	173 (100%)		
Sex	Female	31 (47.69%)	34 (52.31%)	65 (100%)	2.5642	0.109
	Male	65 (60.19%)	43 (39.81%)	108 (100%)		
	Total	96 (55.49%)	77 (44.51%)	173 (100%)		
Religion	Christian	7 (43.75%)	9 (56.25%)	16 (100%)	3.7217	0.156
	Hindu	84 (58.74%)	59 (41.26%)	143 (100%)		
	Muslim	5 (35.71%)	9 (64.29%)	14 (100%)		
	Total	96 (55.49%)	77 (44.51%)	173 (100%)		
Occupation	Employed	76 (53.90%)	65 (46.10%)	141 (100%)	0.7809	0.377
	Unemployed	20 (62.50%)	12 (37.50%)	32 (100%)		
	Total	96 (55.49%)	77 (44.51%)	173 (100%)		
Education	Illiterate	18 (60.00%)	12 (40.00%)	30 (100%)	0.2987	0.585
	Literate	78 (54.55%)	65 (45.45%)	143 (100%)		
	Total	96 (55.49%)	77 (44.51%)	173 (100%)		
Marital status	Married	90 (56.25%)	70 (43.75%)	160 (100%)	0.4962	0.481
	Other	6 (46.15%)	7 (53.85%)	13 (100%)		
	Total	96 (55.49%)	77 (44.51%)	173 (100%)		
Socio-economic status	1,2,3	31 (50.82%)	30 (49.18%)	61 (100%)	0.8326	0.362
	4,5	65 (58.04%)	47 (41.96%)	112 (100%)		
	Total	96 (55.49%)	77 (44.51%)	173 (100%)		
Family history of diabetes	Absent	48 (66.67%)	24 (33.33%)	72 (100%)	6.2360	0.013
	Present	48 (47.52%)	53 (52.48%)	101 (100%)		
	Total	96 (55.49%)	77 (44.51%)	173 (100%)		
Number of years of diabetes	<5 years	69 (53.08%)	61 (46.92%)	130 (100%)	1.2344	0.267
	>5 years	27 (62.79%)	16 (37.21%)	43 (100%)		
	Total	96 (55.49%)	77 (44.51%)	173 (100%)		
Treatment modality	Non-pharmacological	3 (33.33%)	6 (66.67%)	9 (100%)	1.8873	0.170
	Pharmacological	93 (56.71%)	71 (43.29%)	164 (100%)		
	Total	96 (55.49%)	77 (44.51%)	173 (100%)		
Co-morbidities	Absent	82 (60.74%)	53 (39.26%)	135 (100%)	6.8572	0.009
	Present	14 (36.84%)	24 (63.16%)	38 (100%)		
	Total	96 (55.49%)	77 (44.51%)	173 (100%)		

A stronger association emerged with family history of diabetes: participants with a diabetic relative practised better foot care (53, 52.48%) than those without such a history (24, 33.33%), a statistically significant difference (p < 0.05).

Disease-related factors also mattered. Patients within the first five years of diagnosis were more consistent with foot care (61, 46.92%), compared to those diagnosed longer ago (16, 37.21%). Likewise, participants with co-morbidities like hypertension, dyslipidaemia, and ischemic heart disease were markedly more attentive (24, 63.16%) than those without (53, 39.26%), and this relationship was also statistically significant (p < 0.05).

## Discussion

This study, conducted in a rural region of Andhra Pradesh, sheds light on the silent but substantial burden of poor foot health as a sequelae of peripheral neuropathy secondary to diabetes mellitus. Local cultural practices (e.g., barefoot walking within and outside the household), limited use of protective footwear, and carbohydrate-dense diets plausibly amplify foot risk. In instances where BMI/diet data were not captured, we note this as an area for future data collection. The findings point to an urgent need for structured diabetic foot screening and patient education in primary care, particularly in underserved rural settings.

Peripheral neuropathy symptoms were alarmingly common in our population, with the most frequent being impaired temperature sensation (153, 88.44%), followed by muscle cramps, dry skin, and numbness. These figures are comparable to those reported by D’Souza et al. [6] in Karnataka, who found neuropathic symptoms in over half of their study population. The high burden in our study may reflect delayed detection and lack of routine screening at the primary care level. Additionally, the presence of co-morbidities like hypertension, dyslipidaemia, and ischemic heart disease in three out of four participants underscores the multi-system involvement typical of advanced, poorly controlled diabetes.

The predominance of glucose random blood sugar (GRBS) reflects local access constraints. Notably, only 29 (16.76%) of patients had undergone HbA1c testing in the previous year, a striking indicator of limited access to comprehensive glycaemic monitoring in rural settings. This is consistent with earlier observations by Pati and Schellevis [7], who highlighted the gaps in care continuity in public healthcare infrastructure in Eastern India. Despite this, a relatively high percentage (164, 94.78%) were on pharmacological treatment, paralleling national-level estimates reported by Mohan et al. [8] from the A1chieve study.

The foot-care practices observed in this study were suboptimal in several aspects. More than half of the participants did not apply moisturiser or inspect their feet regularly. Only three (1.73%) used a mirror to inspect the soles of their feet - a simple yet powerful preventive strategy. These numbers are consistent with previous studies from South India [9] and Iraq [10], both of which attributed poor practices to low awareness, inadequate counselling, and lack of emphasis on foot care during clinical consultations.

Notably, our analysis revealed that individuals with a family history of diabetes had significantly better foot-care behaviours. This finding supports the notion that greater familial exposure may lead to heightened awareness, vigilance, and more health-seeking behaviours [11,12]. The positive association between family history and better practices suggests experiential learning; structured community awareness campaigns could replicate these gains among those without such exposure. Similarly, those with co-morbidities like hypertension and cardiovascular disease were significantly more likely to engage in protective foot care (p = 0.009), perhaps due to increased interaction with healthcare providers and heightened disease literacy.

Age, gender, literacy, occupation, and socioeconomic class, although traditionally considered important in health behaviour models, did not show statistically significant associations in our analysis. This suggests that while demographic factors play a role, the presence of a supportive health system and strong patient-provider communication may be more critical in driving behavioural change.

To contextualise the findings of the current study, a comparative analysis was conducted with other regional, national, and international studies on diabetic peripheral neuropathy and foot-care practices. Table 5 summarises key characteristics, prevalence of neuropathy symptoms, foot-care awareness levels, and associated observations. This comparison helps highlight common challenges faced in low-resource settings and reinforces the need for structured diabetic foot-care education, especially in rural communities like ours.

**Table 5:** Comparison of Neuropathy and Foot-Care Practices in the Current Study and Other National/International Studies PVD: peripheral vascular disease; VPT: vibration perception threshold

Study (Author, Year, Location)	Sample Size	Neuropathy Symptoms (%)	PVD Prevalence (%)	Good Foot-Care Practices (%)	Notable Observations
Current study (Andhra Pradesh, 2025)	173	88% hot/cold loss, 83.6% numbness, 71% neuropathy	Not calculated	~44.5%	Better foot care is seen with family history and comorbidities. Mirror use is only 1.73%.
D’Souza et al., 2015 (Mangalore, India) [6]	400	53% (burning, numbness, tingling)	-	36%	Neuropathy worsened with poor glycaemic control and age >40.
Saber and Daoud, 2018 (Iraq) [10]	310	55.2%	-	41.6%	Females showed better awareness; low mirror use was reported.
Chiwanga and Njelekela, 2015 (Tanzania) [13]	400	48.2% (based on VPT + symptom screen)	8.7%	30%	Poor footwear practices and poor awareness of foot inspections.
Geetha et al., 2017 (Tamil Nadu, India) [11]	386	78% (symptomatic neuropathy)	-	-	High prevalence of symptoms but limited preventive practice.
Darshan et al., 2015 (Coastal South India) [9]	380	-	-	39.2%	Gender and education significantly influenced care.
Mohan et al., 2013 (A1chieve India Study) [8]	20,554	-	-	-	93.6% on therapy, but no structured foot-care intervention was noted.
Dhandapani et al., 2022 (Rural India) [14]	260	~71% neuropathic symptoms	-	~40%	Rural gaps in awareness; compliance improved with education.
Pati and Schellevis, 2017 (Urban Odisha, India) [7]	1200	-	-	-	Co-morbidities prevalent; health system gaps stressed.
Basu et al., 2005 (West Bengal, India) [15]	240	60% sensory loss	12%	34%	Illiteracy was a major determinant of poor foot hygiene.
Boulton et al., 2004 (Europe/UK - Intl. Guidelines) [16]	-	~50% (diabetic sensory neuropathy)	~10-15%	-	Emphasized education, protective footwear, and daily inspection.

The neuropathy symptom burden in the present study (153, 88.44% hot/cold discrimination loss; 88, 50.87% numbness) is comparable to or slightly higher than rates reported in several Indian and international studies. For example, D’Souza et al. [6] reported 53% neuropathy in Mangalore, while Chiwanga and Njelekela [13] in Tanzania reported 48.2%. Similar to Iraq [10] and South India [9], this study confirms that mirror use and moisturising remain extremely low in rural settings. Unlike some international findings where education strongly impacted practices, in our study, education and occupation were not significantly associated, suggesting cultural and systemic influences are also at play.

Our findings align with international guidelines that emphasise routine foot screening and patient education as central to diabetes care [16]. In Tanzania, Chiwanga et al. [13] found that less than a third of diabetics practised daily foot inspection, and, similar to our findings, insulin users were at increased risk for complications, possibly due to more severe disease. Similar patterns of neuropathy and foot-care gaps were reported in rural West Bengal by Basu et al. [15]. The resemblance of challenges across geographies, India, Iraq, and Tanzania, highlights that diabetic foot care remains a globally underemphasized domain, especially in low and middle-income countries.

Taken together, this study highlights a critical gap in the prevention of diabetic foot complications. Peripheral neuropathy is often asymptomatic or under-recognised, and inadequate foot care accelerates the risk of ulcers and amputations. We recommend village-level counselling, visual infographics, footwear subsidy programs, and periodic mobile-clinic screening to address gaps identified in this study, and simple interventions like educating patients about proper footwear, moisturisation, and daily inspection can drastically reduce complications and healthcare costs. At point-of-diagnosis, providers can deliver brief foot-care counselling with illustrated leaflets, quarterly nurse-led foot checks, referral pathways for footwear fitting, and SMS reminders for follow-up and HbA1c testing.

## Conclusions

This community-based study highlights the high burden of peripheral neuropathy and the widespread gap in preventive foot-care practices among rural adults with T2DM. Peripheral vascular assessment was limited to pulse palpation and, therefore, reported qualitatively. The findings underscore the urgent need for early screening, patient education, and targeted interventions at the primary care level to reduce the risk of preventable complications.

Strengthening rural healthcare services, improving access to diagnostic tools, and integrating culturally appropriate health education into routine diabetic care could significantly improve outcomes. Future studies with larger sample sizes and longitudinal follow-up are warranted to further explore risk factors and evaluate the impact of intervention strategies in similar settings.

## Appendices

Appendix A

General Details

01. Name:

02. Age:

03. Sex: ☐ Male ☐ Female ☐ Other

04. Name of the Village:

05. Phone Number:

06. Religion: ☐ Hindu ☐ Christian ☐ Muslim ☐ Other: _______

07. Occupation: ☐ Unemployed ☐ Agriculture ☐ Unskilled ☐ Semi-skilled ☐ Skilled ☐ Clerical/shop owner ☐ Semi-professional ☐ Professional ☐ Housewife ☐ Retired ☐ Student

08. Education: ☐ Illiterate ☐ Primary ☐ Middle ☐ High ☐ Intermediate ☐ Graduate

09. Total family income per month: ☐ ₹500-5000 ☐ ₹5001-10000 ☐ ₹10001-15000 ☐ ₹15001-20000 ☐ >₹20000 ☐ No income

10. Total family members:

11. Marital status: ☐ Married ☐ Unmarried ☐ Divorced ☐ Separated ☐ Widow/Widower

Diabetic History

Number of years of diabetes: ☐ <1 year ☐ 1-5 years ☐ 5-10 years ☐ >10 years

Treatment modality: ☐ Oral hypoglycemic ☐ Insulin ☐ Both ☐ Diet management ☐ None

Any comorbidities? ☐ Yes ☐ No (Other: ________)

Family history of diabetes: ☐ Absent ☐ Maternal ☐ Paternal ☐ Siblings

HbA1c (if done): Date ___ / ___ / ___ | Value: ☐ <5.7 ☐ 5.7-6.4 ☐ >6.4

FBS (if done): Date ___ / ___ / ___ | Value: ☐ <100 ☐ 100-125 ☐ 126-200 ☐ 201-300 ☐ >300 mg/dl

GRBS (if done): Date ___ / ___ / ___ | Value: ☐ <140 ☐ 141-200 ☐ 200-300 ☐ >300 mg/dl

PPBS (if done): Date ___ / ___ / ___ | Value: ☐ <140 ☐ 140-200 ☐ 201-300 ☐ >300 mg/dl

Observation Checklist: Foot-Care Practices

Do you wash your legs/feet frequently? ☐ Does not wash ☐ Washes with lukewarm water ☐ Washes with hot water

Do you wipe your legs/feet after washing? ☐ Does not wipe ☐ Does not wipe web spaces ☐ Wipes web spaces

Do you apply moisturizer to your feet regularly? ☐ Does not apply ☐ Leaves out web spaces ☐ Applies in web spaces

Do you use a mirror to check feet for unseen areas? ☐ Does not use mirror ☐ Uses correctly

Are you careful while cutting nails? ☐ Does not cut nails ☐ Cuts straight carefully ☐ Not careful

Do you choose footwear based on diabetic condition? ☐ Incorrect footwear ☐ Correct footwear ☐ No idea footwear matters

Screening for Neuropathy

(Answer YES or NO for each)

1. Do your legs/feet feel numb?

2. Do you ever have burning pain in your legs/feet?

3. Are your feet too sensitive to touch?

4. Do you get muscle cramps in your legs/feet?

5. Do you ever have a prickling feeling in your legs/feet?

6. Does it hurt when the bed covers touch your skin?

7. When in the shower, can you tell hot from cold water?

8. Have you ever had an open sore on your foot?

9. Has your doctor told you that you have diabetic neuropathy?

10. Do you feel weak all over most of the time?

11. Are your symptoms worse at night?

12. Do your legs hurt when you walk?

13. Are you able to sense your feet when you walk?

14. Is the skin on your feet so dry it cracks?

15. Have you ever had an amputation?

Physical Screening for Neuropathy

Appearance of feet: ☐ Normal ☐ Dry/cracked ☐ Surgical scars ☐ Amputated ☐ Wounds/injuries ☐ Not clean

Ulceration: ☐ Absent ☐ Present & healing ☐ Present & non-healing

Ankle reflex: ☐ Absent ☐ Present

Vibration perception at great toe: ☐ Present & normal ☐ Reduced ☐ Absent

Monofilament test: ☐ Present & normal ☐ Reduced ☐ Absent

Peripheral Pulses

Right dorsalis pedis artery: ☐ <40 bpm ☐ 40-60 ☐ 60-80 ☐ 80-100 ☐ 100-120 ☐ >120

Left dorsalis pedis artery: ☐ <40 bpm ☐ 40-60 ☐ 60-80 ☐ 80-100 ☐ 100-120 ☐ >120

Right posterior tibial artery: ☐ <40 bpm ☐ 40-60 ☐ 60-80 ☐ 80-100 ☐ 100-120 ☐ >120

Left posterior tibial artery: ☐ <40 bpm ☐ 40-60 ☐ 60-80 ☐ 80-100 ☐ 100-120 ☐ >120
